# Supplementary material for: Procymidone Application Contributes to Multidrug Resistance of Botrytis cinerea
Source: J Fungi (Basel). 2024 Mar 29;10(4):261. doi: 10.3390/jof10040261 (PMC11050779; doi:10.3390/jof10040261)
Supplement: Supplementary file 1 [file jof-10-00261-s001.zip › jof-2927202-supplementary.pdf]

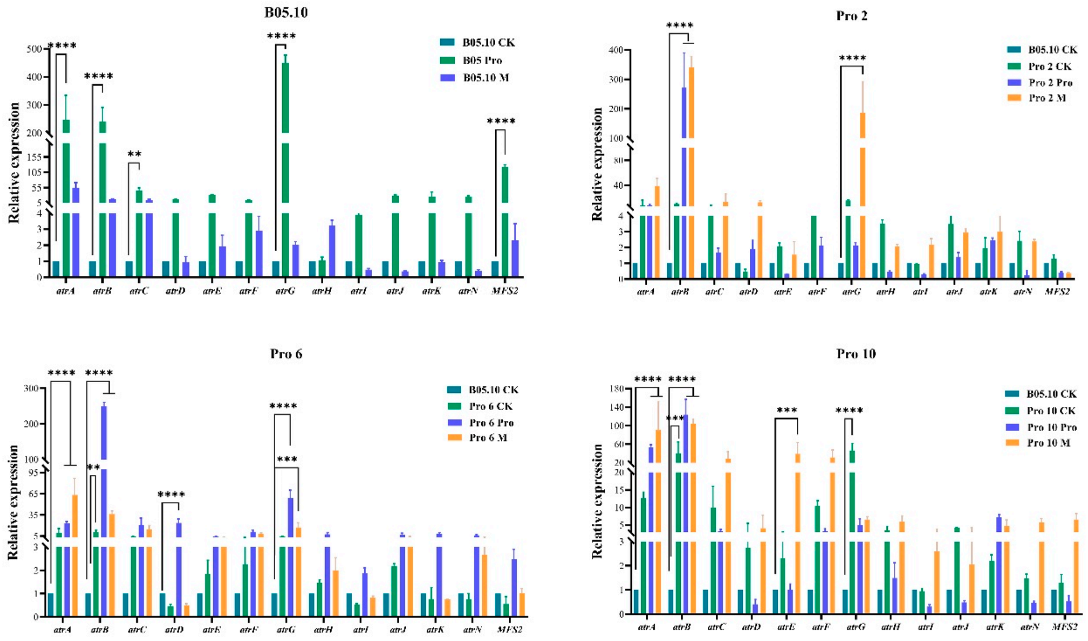

Supplement Figure S1. Quantitative polymerase chain reaction (qPCR) analysis of 14 ABC transporter genes in procymidone-resistant mutants with *BcEF* being a reference gene. CK: non-treated; Pro: procymidone added; M: azoxystrobin added; '****' indicates *p* value < 0.0001; '***' indicates *p* value < 0.001; '**' indicates *p* value < 0.001.

Supplement Table S1. Primers used for sequence analysis.

Genes	Sequences
BcBos1	F: ACATCACAATGTCGACATGGCAGCCATTGAGCCTG<br>R: CCTTGCCCATTCTAGATTCAGCCCCCTTGGTCTTC
SdhA	F: ATTTCTACACAATGTCTTCATTTGC<br>R: ATCATACTAATCCGCTCC
SdhB	F: ACCTACTCGCCCTATCCAAT<br>R: AGACTTAGCAATAACCGCCC
SdhC	F: GCCAGATTTCCTTAGTCAG<br>R: GCTGGACTCTGAATGTGAT
SdhD	F: AGCCAATCAAATCCGTTCCG<br>R: CAAACTCCTCCCTGCCCTCT
Cytb	F: TAAAGTGGTATAACCCGACGG<br>R: CCATCTCCATCCACCATACCT
Mrr1	F: CCAATCATTCCCAATCATTCA<br>R: GGATAGGGTATTGCGTAGATCG

F: forward primer. R: reverse primer.
